# Dry Matter Gains in Maize Kernels Are Dependent on Their Nitrogen Accumulation Rates and Duration during Grain Filling

**DOI:** 10.3390/plants10061222

**Published:** 2021-06-15

**Authors:** Lía B. Olmedo Pico, Tony J. Vyn

**Affiliations:** Department of Agronomy, Purdue University, 915 West State Street, West Lafayette, IN 47907, USA; lolmedop@purdue.edu

**Keywords:** kernel weight, grain-filling duration, effective grain-filling rate, kernel N content, kernel N accumulation duration, kernel N accumulation rate, nitrogen nutrition index

## Abstract

Progressive N assimilation by maize kernels may constrain dry matter (DM) accumulation and final kernel weights (KW). We sought to better understand whole-plant and kernel N mechanisms associated with incremental DM and N accumulation patterns in kernels during grain fill. Maize was grown with multiple fertilizer N rates and N timings or plant densities to achieve a wide N availability gradient. Whole-plant DM and N sampling enabled determination of apparent N nutrition sufficiency at flowering (NNI_R1_) and when linear-fill began (NNI_R3_). Linear-plateau, mixed-effects models were fitted to kernel DM and N accumulation data collected weekly from early R3. Higher N supply, regardless of application timing or plant density, increased grain-fill duration (GFD) and, more inconsistently, effective grain-filling rate (EGFR). Kernels accumulated DM and N for similar durations. Both final KW and kernel N content increased consistently with N availability mostly because of higher kernel N accumulation rates (KNAR) and duration (KNAD). Both NNI_R1_ and NNI_R3_ were positively associated with KNAD and KNAR, and less strongly with EGFR. These results confirm the direct role of kernel N accumulation, in addition to prior NNI, in limiting KW gain rates and duration during grain filling.

## 1. Introduction

Maize grain yield is defined by the product of kernel number (KN) and kernel weight (KW). Although KN is considered the main grain yield determinant [1,2,3] because it is more responsive to changes in environmental conditions [4,5,6,7], grain yield can still be affected by variations in KW [8]. In terms of genetics, as a result of breeding improvement in sink strength, modern genotypes have shown more KW variation than their older counterparts [9,10]. Additionally, recent studies with current maize hybrids reported that final KW was proportionally more responsive than KN, and thus more closely related to grain yield variations, under differences in planting dates [11,12]. Therefore, the prospect of KW becoming a relatively more important driver behind in-field grain yield variability warrants a closer look into the physiological mechanisms that play a role when post-flowering stress conditions limit KW.

Final KW is the result of physiological processes taking place throughout the grain-filling period (i.e., from silking to maturity). Once potential KW is defined through endosperm cell division (with negligible dry weight gain) in the lag phase [13,14], kernels then enter the effective grain-filling phase, where they actively accumulate dry matter (DM) at a constant rate until reaching physiological maturity [15]. Both the rate and the duration of DM accumulation (i.e., effective grain-filling rate -EGFR-, and grain-filling duration -GFD-, respectively) thus constitute the determining factors of final KW [15,16]. In addition, around mid-filling, kernel water content increases to reach a maximum and then it decreases as dry matter accumulation continues [17,18]. Both maximum kernel water content and EGFR are also related to potential KW [13,19]. Whether kernels achieve this previously established potential size has previously been reported to depend on the availability of assimilates per kernel (i.e., source–sink relationship) during the linear phase [3,5]. The main source of assimilates for the growing kernels is current net photosynthesis (estimated as plant growth gain during grain filling) [3,20], while stem reserves play a role when sink demands are higher than photosynthetic source capacity [21,22].

Individual KW is a genetically determined trait [23], and it varies among genotypes via different combinations of EGFR and GFD [24,25]. However, environmental conditions during the linear phase, such as extreme temperatures [26] or water stress [27], also change final KW. Furthermore, at the crop level, decreases in KW under low N conditions have been associated with reductions in post-silking biomass accumulation (i.e., photosynthetic source capacity) and with changes in source–sink ratio [21,28]. On a per-kernel basis, DM dynamics have been described for numerous experimental conditions, but few studies have investigated how kernel growth parameters (i.e., GFD and EGFR) might be affected by contrasting N availability scenarios. For example, KW changes under different N supply have been related to changes in grain-filling rate [29] or changes in both the EGFR and the GFD [30,31]. However, because maize response to N is often a function of N timing and plant density, further research into the physiological determinants of final KW under a wide per-plant and per-kernel N availability gradient could help identify underlying mechanisms and management combinations for achieving higher KW.

While carbohydrates dominate the DM accumulated by kernels during grain filling, N assimilates are also actively demanded by these sink tissues [32]. N allocated to the kernels comes from post-silking N uptake and/or N remobilization from leaves and stems [33,34], and it is used for the synthesis of both storage proteins and enzymes required to convert soluble sugars and amino acids into starch and proteins, respectively [35,36]. Despite its crucial role in achieving final KW, kernel N accumulation over time has not been studied in the same detail as DM. Recent studies have looked into kernel N accumulation from a descriptive perspective by plotting linear interpolations between five [37] or seven [38] consecutive sampling dates over the grain-filling period. While both of these studies provided valuable insight into kernel N dynamics, the N availability ranges were limited to 2–3 N rates (without a secondary factor), and the lack of kernel samplings beyond 50–58 days after silking (DAS) prevented a deeper analysis of possible peaks or plateaus that may further explain the relationship of final KW with final kernel N content. Furthermore, the dependency of kernel biomass versus N accumulation patterns on whole-plant N sufficiency (as quantified in the nitrogen nutrition index - NNI) [39] before and at the beginning of linear fill is also unknown. Given the tight interactions between C and N source–sink dynamics [40,41], describing kernel N accumulation via well-known characterization parameters should help in better understanding physiological mechanisms associated with DM accumulation, and ultimately final KW.

To the best of our knowledge, there have been no prior studies focused on maize kernels that sequentially determined both DM and N dynamics at weekly time intervals under a wide range of in-field N availability conditions. Therefore, the objectives of this work were: (1) to study kernel DM and N kernel dynamics during the linear phase of grain filling following whole-plant DM and N assessments, (2) to determine N rate effects on the parameters thus obtained and (3) to study the relationships between the parameters underlying the incremental kernel N versus DM gains during grain fill. 

## 2. Results

### 2.1. Grain Yield, Kernel Number per Plant, Kernel Weight and Final Kernel N Content

GY, its components, and grain N content (KNC) at R6 consistently responded to N rate treatments across all experiments (Table 1, Table 2 and Table 3), without showing significant interactions with either application timing (Experiment 1) or plant density (Experiments 2 and 3). While N application timing main effects were never significant in Exp. 1, plant density sub-treatment effects were detected in some parameters in Exp. 2 and 3. However, KW was only affected by density in Exp. 2 (Table 2). The highest gains in GY (~10.4 Mg ha^−1^), KW (~100 mg grain^−1^), and KNC (2.4 mg N grain^−1^) from 0 N to 224 N occurred in Exp. 1 (Table 1). Although the highest treatment means of both GY and KW in Exp. 1 and Exp. 2 were similar (15.7 and 16.6 Mg ha^−1^, respectively), the GY and KW means did not decline as much under 0 N in the latter study (Table 2), probably due to a bigger contribution from soil mineralization. Conversely, though Exp. 3 was conducted in the same location (but not in the same field) as Exp. 2, the significant delay in planting constrained the realization of the hybrid’s GY potential by lowering both KNP and KW (Table 3). Despite these seasonal differences, average GY increased 8.1 Mg ha^−1^ in response to N (i.e., from 0 N to 224 N) over the 3-year period. While kernel N concentrations (KNc) were similar in Exp. 2 and 3, lower KNC values were observed across N rates in Exp. 3 due to a lower overall KW.

### 2.2. Plant Growth and N Uptake during the Effective Grain-Filling Period

As expected, total-plant DM production and N uptake at the onset of the effective grain-filling period (PG_R3_ and PNU_R3_, respectively), as well as total-plant DM and N accumulation over the whole effective grain-filling period (PG_R3_._R6_ and PNU_R3_._R6_), responded positively to soil N availability in all experiments (Table 1, Table 2 and Table 3), except for PNU_R3_._R6_ in the lower yielding Exp. 3. In addition, interaction effects between application timings and N rate (Exp. 1) or between N rate and plant density (Exp. 2 and 3) were never significant. While in Exp. 1 the timing of fertilizer application did not impact PG_R3_, PNU_R3_, PG_R3_._R6_ and PNU_R3_._R6_ (Table 1), most of these parameters were reduced at higher plant density in Exp. 2 (i.e., PG_R3_, PNU_R3_ and PG_R3_._R6_, Table 2) and Exp. 3 (i.e., PG_R3_ and PNU_R3_, Table 3).

In terms of photosynthetic source capacity, PG_R3_._R6_ reached similar maximum values in Exp 1 and 2, regardless of lower starting biomass (i.e., at R3) in the latter (Table 1 and Table 2). Under higher N rates, Exp. 3 produced just half the PG_R3_._R6_ (compared to Exp. 2) while having similar R3 biomass (Table 2 and Table 3). Under increases in N rate, both PNU_R3_ and PNU_R3_._R6_ gains were greater in Exp. 1 (1.52 and 0.81 g N plant^−1^, respectively), while being lower in Exp. 3 (0.96 g N plant^−1^ and non-significant, respectively). The remarkably lower biomass production and N uptake present in Exp. 3 were consistent with a less favorable growing season due to delayed planting.

### 2.3. Nitrogen Nutrition Index at Silking and at the Onset of the Linear Phase

In the three experiments, the majority of NNI values at silking were larger than those achieved at the onset of the linear grain fill (Table 1, Table 2 and Table 3). As expected, NNI at both reproductive stages was highly impacted by N rate treatments, with no significant effects from N timing application (Exp. 1), plant density (Exp. 2 and 3), nor their respective interactions with N rate (Table 1, Table 2 and Table 3). Additionally, average NNI values at R3, regardless of N rate, were consistently below 1.0. The smallest proportional reductions in NNI from R1 to R3 (for the same N rates) was observed in Exp. 2, while R1-R3 NNI decreased most in Exp. 1.

### 2.4. Dry Matter Accumulation Dynamics in Kernels

**Figure 1:**
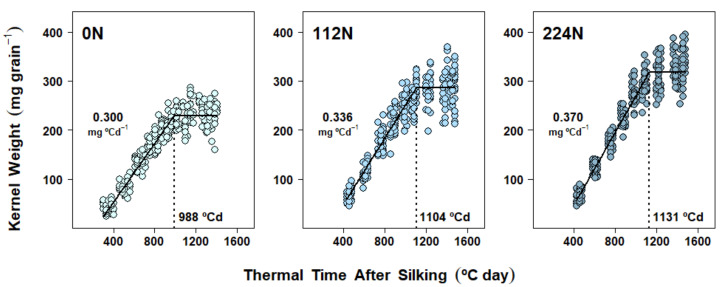
Dry matter accumulation in maize kernels in Experiment 1 (La Crosse, 2017). Each panel shows data obtained from plants grown under one N rate (0, 112, 224 kg N ha^−1^) applied at three different application timings. Each point represents the average of 15 kernels sampled from the same ear. Full lines represent the N rate effect on the fitted linear-plateau model. Grain-filling duration (GFD) is pointed by the dotted vertical line. Effective grain-filling rate (EGFR) is shown above the points.

**Figure 2:**
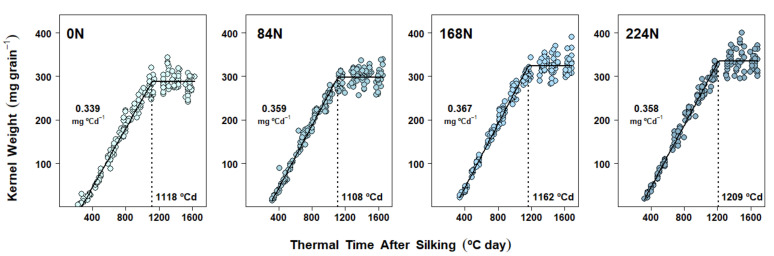
Dry matter accumulation in maize kernels in Experiment 2 (West Lafayette, 2018). Each panel shows data obtained from plants grown under one N rate (0, 84, 168, 224 kg N ha^−1^) at two different plant densities. Each point represents the average of 15 kernels sampled from the same ear. Full lines represent the N rate effect on the fitted linear-plateau model. Grain-filling duration (GFD) is pointed by the dotted vertical line. Effective grain-filling rate (EGFR) is shown above the points.

**Figure 3:**
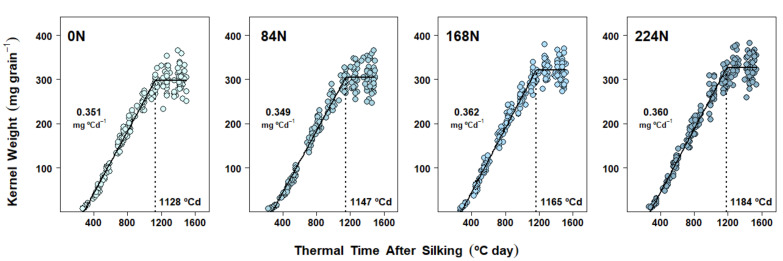
Dry matter accumulation in maize kernels in Experiment 3 (West Lafayette, 2019). Each panel shows data obtained from plants grown under one N rate (0, 84, 168, 224 kg N ha^−1^) at two different plant densities. Each point represents the average of 15 kernels sampled from the same ear. Full lines represent the N rate effect on the fitted linear-plateau model. Grain-filling duration (GFD) is pointed by the dotted vertical line. Effective grain-filling rate (EGFR) is shown above the points.

Kernel DM accumulation under the different N rate treatments resulted from either a combination of changes in both the effective grain filling rate (EGFR) and the grain filling duration (GFD) (Exp. 1 and Exp. 2, Figure 1 and Figure 2, Supplementary Tables S1 and S2, respectively) or changes in GFD only (Exp. 3, Figure 3, Supplementary Table S3). Averaging across N rate treatments, maize reached maximum kernel weights at 1074 °C day in 2017, 1149 °C day in 2018, and 1156 °C day in 2019, which approximately corresponded with 55, 51, and 53 days after silking, respectively.

In Exp. 1, the major factor affecting KW variability was N rate, regardless of when the fertilizer was applied (Table 1). The application timing fixed effect was tested within the mixed-effect model and, though convergence was achieved, it did not increase the predictive capacity of the model due to its lack of significance on the parameters. Thus, we pooled data from the three application timings to run a mixed-effect, nonlinear model analysis using N rate alone (Figure 1). DM accumulation by kernels showed three distinct patterns, depending on the amount of total N that plants received throughout the growing season, because of positive changes in both EGFR and GFD. EGFR increased gradually with N supply: 12% from 0 N to 112 N (*p* < 0.0001), 10% from 112 N to 224 N (*p* < 0.0001), and 23% from 0 N to 224 N (*p* < 0.0001) (Figure 1 and Table S1). Additionally, N supply increased GFD by 116−143 °C day (*p* < 0.0001) (approximately similar to an actual 8- to 10-day increase under these climatic conditions), but no GFD differences were found between 112 N and 224 N (*p* = 0.49) (Figure 1 and Table S1).

When the mixed-effect nonlinear model was applied to Exp. 2 and Exp. 3 datasets, plant density fixed effects were ultimately excluded due to lack of convergence and, less frequently, zero improvement (or worsening) of the goodness of fit. Therefore, the final models for DM accumulation dynamics were analyzed in terms of the N rate fixed effects only. N rate effects on EGFR were significant in Exp. 2, where fertilized treatments averaged 7% higher EGFR vs 0 N (all three contrasts with *p* < 0.01) (Figure 2 and Table S2), while EGFR was rather consistent across N rates in Exp. 3 (Figure 2 and Supplementary Table S3). Significant differences in GFD were detected in both Exp. 2 and Exp. 3. A more gradual increase in GFD due to N rate was evident in Exp. 2 (Figure 2 and Table S2) and although GFD was not different from 0 N to 168 N in Exp. 3, it increased under 224 N (*p* = 0.05) (Figure 3 and Supplementary Table S3).

### 2.5. Nitrogen Accumulation Dynamics in Kernels

**Figure 4:**
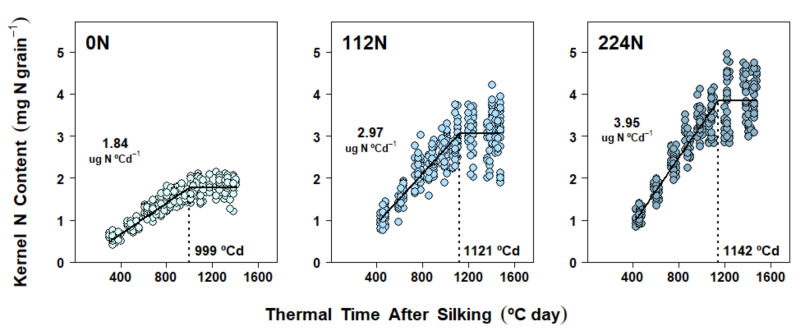
N accumulation in maize kernels in Experiment 1 (La Crosse, 2017). Each panel shows data obtained from plants grown under one N rate (0, 112, 224 kg N ha^−1^) applied at three different application timings. Each point represents the average of 15 kernels sampled from the same ear. Full lines represent the N rate effect on the fitted linear-plateau model. Kernel N accumulation duration (KNAD) is pointed by the dotted vertical line. Kernel N accumulation rate (KNAR) is shown above the points.

**Figure 5:**
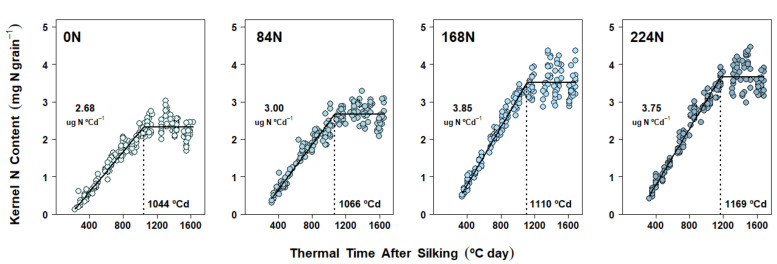
N accumulation in maize kernels in Experiment 2 (West Lafayette, 2018). Each panel shows data obtained from plants grown under one N rate (0, 84, 168, 224 kg N ha^−1^) at two different plant densities. Each point represents the average of 15 kernels sampled from the same ear. Full lines represent the N rate effect on the fitted linear-plateau model. Kernel N accumulation duration (KNAD) is pointed by the dotted vertical line. Kernel N accumulation rate (KNAR) is shown above the points.

**Figure 6:**
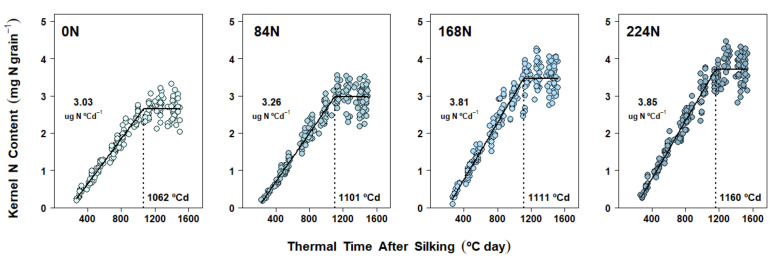
N accumulation in maize kernels in Experiment 3 (West Lafayette, 2019). Each panel shows data obtained from plants grown under one N rate (0, 84, 168, 224 kg N ha^−1^) at two different plant densities. Each point represents the average of 15 kernels sampled from the same ear. Full lines represent the N rate effect on the fitted linear-plateau model. Kernel N accumulation duration (KNAD) is pointed by the dotted vertical line. Kernel N accumulation rate (KNAR) is shown above the points.

Kernel N accumulation followed a pattern similar to that of grain DM accumulation, with an active, linear import of N assimilates for most of the grain-filling period and then a plateau near physiological maturity in all three experiments (Figure 4, Figure 5 and Figure 6). Furthermore, differences in kernel N accumulation dynamics were always realized through changes in both kernel N accumulation rate (KNAR) and kernel N accumulation duration (KNAD). In Exp. 1, N accumulation was once again studied considering only N rate fixed effects due to the lack of application timings impacts on final KNC (Table 1). Increases in N rate produced a similar KNAD variation as that found for GFD, while KNAR response reflected a much bigger relative gain (compared to EGFR) with higher N supply: 61% from 0 N to 112 N (*p* < 0.0001), 33% from 112 N to 224 N (*p* < 0.0001), and 115% from 0 N to 224 N (*p* < 0.0001) (Figure 4 and Table S4).

In Exp. 2 and Exp. 3, N rate fixed effects on kernel N accumulation dynamics were also studied without including plant density effects. Kernel N accumulation patterns detected in these two experiments were somewhat similar to those of Exp. 1, with N supply increasing final KNC by changing both KNAR and KNAD (Supplementary Tables S5 and S6). While relative gains in the parameters were different from season to season, a common pattern of KNAR increasing gradually until 168 N was found in both experiments. In Exp. 2, KNAR showed a 12% increase from 0 N to 84 N (*p* = 0.0001), a 28% increase from 84 N to 168 N (*p* < 0.0001), and a 44% increase from 0 N to 168 N (*p* < 0.0001) (Figure 5 and Table S5). In Exp. 3, KNAR increased by 8% from 0 N to 84 N (*p* = 0.03), by 17% from 84 N to 168 N (*p* < 0.0001), and by 26% from 0 N to 168 N (*p* < 0.0001) (Figure 6 and Supplementary Table S6). Kernel N accumulation plateaued at similar thermal times from 0 N to 168 N in Exp. 2, but KNAD increased significantly with 224 N (Figure 5 and Supplementary Table S5). In turn, significant gains in KNAD in Exp. 3 were only detected from 0 N to 224 N (Figure 6 and Supplementary Table S6).

### 2.6. Relationships between Parameters

To examine relationships between variables, pairwise Pearson’s correlation analysis was applied among the DM and N grain-filling parameters estimated by nonlinear regression and the N rate means of GY, KNP, KW, KNC, PG_R3_, PG_R3_._R6_, PNU_R3_, PNU_R3_._R6_, NNI_R1_, and NNI_R3_ (Table 4). As expected, GY was positively correlated (to different degrees) with all variables. The parameters that were highly correlated with KW, but not at all significantly with final KNP, included EGFR and GFD. Final KW was slightly more highly associated with EGFR than with GFD. Final KNC was similarly correlated with both KNAD and KNAR. 

Given the similarities between kernel DM and N allocation dynamics, GFD and KNAD were strongly correlated (Table 4), further supporting the fact that N was actively imported alongside carbohydrate assimilates by kernels until late in the reproductive period. The association between EGFR and KNAR was also highly significant, with the correlation being somewhat higher than that of their respective durations. Interestingly, final KW showed a strong, high association with both KNAR and KNAD, suggesting that N flux to the kernel during the linear period can limit the realization of final KW (Table 4). 

In terms of whole-plant parameters, PNU_R3_._R6_ was not related to any of the DM or N grain-filling parameters (except for KNAD), while EGFR, KNAD, and KNAR were all significantly associated with PNU_R3_ (Table 4). Furthermore, EGFR and GFD were not associated with PG_R3_, but both increased with PG_R3_._R6_. Additionally, both NNI at R1 and at R3 were highly correlated with the whole-plant N uptake that followed (PNU_R3_._R6_) and with kernel N accumulation parameters (i.e., KNAR and KNAD). Conversely, NNI_R1_ was only associated with EGFR, while NNI_R3_ was significantly related to both EGFR and GFD (Table 4). This could also explain the fact that final KW (and even GY) was more highly correlated with NNI at R3 than at R1.

## 3. Discussion

Given the increasing relevance of KW variability in explaining GY limitations in modern maize genotypes [10,11,42,43], a comprehensive study was conducted in order to better understand how soil N availability affected this yield component. An intensive ear sampling was performed for 9−10 weeks beginning at 250−300 °C day after silking to estimate KW defining parameters (i.e., effective grain filling rate (EGFR) and grain filling duration (GFD)) [15,44] and compare them among different N supply treatments. Furthermore, kernel N accumulation dynamics were also characterized via similar parameters (i.e., kernel N accumulation N rate (KNAR) and kernel N accumulation duration (KNAD)) in order to determine possible interactions with DM allocation, given that kernels are simultaneously N sink tissues during grain fill [32,45]. Although KW also depends on KN, our analysis (given our intentional early R3 sampling for initial KW) assumed that plants had already reached their final KNP. Final KN in our experiments reflected a similarly strong dependency on NNI status of individual plots at both R1 and R3 stages (Figure S1). This wide-ranging background in KN over N treatments and site-years gave us the opportunity to document the primary drivers of KW attainment from the R3 stage onwards. 

### 3.1. Overview of Responses to Nitrogen Timing and Plant Density Treatments

Across experiments, N rate treatments produced the strongest effects on both DM and N accumulation in kernels (Figure 1, Figure 2, Figure 3, Figure 4, Figure 5 and Figure 6), regardless of: (a) the time when the fertilizer was applied (Exp. 1), (b) the competition for resources by changes in plant density (Exp. 2 and 3), and (c) their respective interactions with N rate (all non-significant, Table 1, Table 2 and Table 3). While late-season, split N applications have been associated with higher N recovery efficiencies due to increased post-silking N uptake [46], split applications explored in Exp. 1 did not produce any benefits in either N content by R3 or post-R3 N uptake when compared to the at-planting N application (Table 1). The latter partially explains the lack of N timing effect found on both final KNC and kernel N accumulation dynamics. The lack of N timing effect on KW (as well as on KNP) represented a more common outcome, as these management practices are less likely to increase final GY [46,47]. While previous research investigated kernel DM progression across split N applications by sampling kernels four times over the grain-filling period [48], grain-filling rate and duration parameters were not reported. Therefore, to the best of our knowledge, no other studies have previously tested kernel DM accumulation parameters such as GFD and EFGR during grain fill under differences in N timing applications alone or combined with N rate.

In terms of plant density effects, usually associated with source–sink balances reflecting assimilate availability for the kernels to grow, decreases in KW by changes in EGFR and/or GFD under higher plant densities (i.e., less resources per kernel) have been well documented [16,17,49]. Nevertheless, in our study, the higher plant density only reduced final KW in Exp. 2, with no effect on KNC (Table 2), while the opposite pattern was found in Exp. 3 (i.e., no effect on KW but reduced KNC under higher plant stand, Table 3). Despite the latter inconsistency, the plant density fixed effect was never included in the final mixed-effects models in either Exp. 2 or 3 (see Materials and Methods), given that the use of simpler models, i.e., those accounting only for N rate fixed effects, were enough to explain the observed variability in kernel DM accumulation dynamics. Conversely, our results differed from a recent report where grain-filling parameters were determined under combinations of three N rates (ranging from 0 to 360 kg N ha^−1^) and two plant densities (6.8 vs 9.8 plant m^−2^) [29]. In that study, KW was changed by plant population, N rate, and their respective interaction, with the effect of density being the strongest, and EGFR proving to be the most affected parameter. Nevertheless, the lack of plant density effects in our results could be explained by the fact that plant density effects on GFD and EGFR can differ proportionally depending on the position in the ear where kernels were collected [50]. In the latter study, much larger decreases in KW due to increases in plant density were registered in kernels coming from the basal and apical ear sections than those coming from the middle (where all our kernel samples were collected). This differential response can be explained by differences in pollination timing along the rachis, where middle-section kernels start growth earlier, thus having the advantage of enough assimilate supply even under limiting conditions [50]. 

The lack of effect of both N timing application and plant density treatments could also be explained by the experimental treatment ranges used in our studies. While our actual density and N timing ranges were similar to current farmer practices in dryland maize systems in Midwest United States, they might have not been extreme enough as to produce significant changes in the kernel DM allocation parameters. 

### 3.2. Nitrogen Availability Effects on Kernel Dry Matter Accumulation Dynamics 

Final KW was significantly increased by N rate in all three experiments (Table 1, Table 2 and Table 3). This strong, consistent response is in line with the concept that modern, high-yielding maize hybrids have more flexible KWs than older genotypes [9,10]. This is further supported by the fact that the hybrid used in our study, commercially released in 2015, achieved high yields of 15.7−16.6 Mg ha^−1^ in two out of three seasons (2017 and 2018, respectively), while averaging an increase of 8.1 Mg ha^−1^ in response to N (i.e., from 0 N to 224 N) over the 3-year period. In addition, GY variability was largely explained by KW in all experiments, as shown by the r = 0.90 from correlation analysis (Table 4).

However, the most novel kernel DM and kernel N findings in our research came about because of the intensive individual kernel sampling during the linear grain-filling period in all three experiments. The subsequent modeling of kernel dynamics of simultaneous DM and N gains in response to N rate treatments provided new clarity on the underlying rate and duration processes. Accordingly, kernel DM accumulation dynamics were demonstrably affected by N rate treatments (Figure 1, Figure 2 and Figure 3). However, depending on the season, two different mechanisms were detected: DM accumulation in kernels responded to N supply either (a) via changes in both EGFR and GFD (Exp. 1 and Exp. 2, Figure 1 and Figure 2, Supplementary Table S1 and Supplementary Table S2, respectively), or (b) via changes in GFD alone (Exp. 3, Figure 3, Supplementary Table S3). Prior research also observed KW changes associated with both EGFR and GFD [31], as we found in Exp. 1 and 2. Our correlation analysis confirmed that both grain fill DM parameters were highly associated with KW, and even though GFD was improved with N rates in all three experiments, EGFR still presented a slightly higher Pearson’s coefficient (Table 4). Similarly, Liu et al. [30] also found that EGFR and GFD were increased under N fertilization, thus enhancing final KW. Furthermore, these authors reported that positive N nutrition effects on KW were even bigger when fertilization included P and K.

EGFR is dependent on potential sink capacity defined earlier in the reproductive season [13,14]. Therefore, changes in this parameter would reflect N treatment effects on potential KW determination established during the lag phase rather than responses to actual growing conditions during the linear period. This could be further supported by the lack of correlations (with PG_R3_ and PNU_R3_._R6_) or the weaker correlations (with PG_R3_._R6_ and PNU_R3_) between EGFR and the whole-plant parameters involving DM and N (Table 4). Once potential KW is set in the lag phase (and, as a consequence, a particular EGFR defined), DM deposition in kernels would depend on the availability of assimilates per kernel during the grain-filling period [3]. Therefore, under higher N supply, indirect N effects on KW via longer GFD could be explained by prolonged leaf N retention (i.e., delayed remobilization) [43,51], a greater post-silking N uptake, and an extended photosynthetic capacity. Modern hybrids are able to retain N longer (i.e., increased leaf area duration) due in part to their greater use of stem N in early reproductive stages [43,52]. In our study, this mechanism was initially supported by the gains detected in all four whole-plant variables under higher N rate: R3 biomass, post-R3 plant growth, R3 N uptake and post-R3 N uptake (Table 1, Table 2 and Table 3). Additionally, another evidence came from the correlation analysis since GFD proved to be associated, though weakly, with both PG_R3_._R6_ and PNU_R3_ (Table 4). 

NNI, an indicator of whether the crop has realized luxury N (NNI > 1), optimum N (NNI ≅ 1), or N deficiency (NN < 1) conditions [39], was not correlated with GFD, but weakly associated with EGFR, when it was estimated at silking (i.e., NNI_R1_, Table 4). Conversely, plant N status estimated at the onset of linear grain fill (i.e., NNI_R3_) was a far better indicator of N rate effects on final KW as it was more strongly related to both EGFR and GFD. Even though NNI_R1_ has been used as an estimator of photosynthetic capacity after silking associated with delayed senescence [53] and increased post-silking N uptake [47], our primary DM related finding was that GFD was predicted by NNI_R3_ but not by NNI_R1_ with this hybrid in these management/environment situations. While we acknowledge that our interesting findings regarding NNI at R3 are insufficient, in themselves, to warrant using this metric as a routine plant diagnostic indicator, the critical N dilution curve in maize is valid until 25 days after silking [54].

### 3.3. Nitrogen Availability Effects on Kernel Nitrogen Accumulation Dynamics 

Final KNC was also strongly affected by soil N supply (Table 1, Table 2 and Table 3), thus mirroring both KW and GY responses. Given the importance of this physiological parameter, abundant research has been done around the sources of KNC [32,34,55,56,57]. However, the dynamics of N accumulation on a per-kernel basis (i.e., KNAD and KNAR) have been less frequently considered in field experiments. Moreover, from the few cases where KNC was determined periodically during the grain-filling period [37,38,58], only one study had formally modeled these data as a function of days after silking [58]. Therefore, to the best of our knowledge, our study represented the first effort to describe KNC over the grain-filling period by estimating KNAD and KNAR characterization parameters on a thermal-time basis. 

Overall, kernel N accumulation followed a pattern similar to that of DM (Figure 4, Figure 5 and Figure 6): kernels actively imported N assimilates for most of the grain-filling period. While kernel N accumulation dynamics were strongly affected by N rate treatments (similarly to DM dynamics), one consistent mechanism was detected across experiments: both parameters (KNAR and KNAD) increased with N rate (Figure 4, Figure 5 and Figure 6; Supplementary Tables S4–S6). Therefore, final KNC was strongly correlated with both KNAD and KNAR (Table 4). 

While post-R3 plant N uptake was not associated with KNAR (and only weakly related to KNAD), both parameters were highly related to whole-plant N accumulated by R3 (Table 4). This is consistent with the fact that plant N status achieved at early reproductive stages (i.e., R1 and R3) proved to be a good indicator of overall kernel N accumulation dynamics later in the linear phase, given that NNI at both R1 and at R3 were highly correlated with KNAR and KNAD. 

### 3.4. Strong Relationships of Kernel Nitrogen and Dry Matter Gains during Linear Grain Fill

A strong association between GFD and KNAD was observed in the correlation analyses between DM and N parameters (Table 4). This supported the fact that N was actively imported by kernels in tandem with carbohydrate assimilates until late in the reproductive period. The association between EGFR and KNAR was also significant, showing a correlation slightly higher than that of the durations (Table 4). The strong relationship between kernel DM and N accumulation rates we observed is consistent with the fact that transport rates of total carbohydrates and amino acids into the endosperm are linearly related during the effective grain-filling period [59].

The tight interaction between kernel DM and N accumulation dynamics was further confirmed by another key finding in our study: final KW was highly correlated with both KNAR and KNAD (Table 4). The latter suggests that the N flux to the kernel during the linear period clearly limits the realization of final KW. This agrees with a similar conclusion (i.e., that kernel C accumulation in N deficient plants might be limited by kernel N availability) reached by Ning et al. [38] because, in their case, kernel C:N ratios increased as final KW dropped under low N supply conditions. Besides the stoichiometric hypothesis proposed by these authors, limitation of DM accumulation in kernels by N deficiency could also be related to the portion of allocated N that is used within the kernel tissues as substrate for synthesis of enzymes [36,45]. The importance of KNAR and KNAD in the realization of final KW uncovered in our study builds on the conclusions found in previous research [60] about the relationship between ear N accumulation rate during early reproductive stages and KW. Furthermore, while N availability effects on KW have usually been related to source–sink balances [21,22,28,61], our findings revealed that N also plays a direct role in DM accumulation in kernels and, therefore, in final KW. Overall, our intensive kernel sampling and later modeling of kernel DM and N accumulation dynamics resulted in a better understanding of the physiological mechanisms behind KW realization as affected by N availability.

## 4. Materials and Methods

### 4.1. Field Experiments

Three experiments were carried out in order to study dry matter and N dynamics in maize kernels. One common Dekalb genotype (hybrid DKC63-60RIB GENSS, commercially released in 2015) was used in the three field studies. Experiment 1 (2017) was conducted at the Purdue Rice Farm (La Crosse, IN), while Experiment 2 (2018) and Experiment 3 (2019) were located at the Purdue Agronomy Center of Research and Education in West Lafayette, IN. All experiments followed a split-plot, randomized complete block design, involving N rate treatments, applied in the form of 28% urea ammonium nitrate (UAN), alongside a secondary factor such as N timing application (Experiment 1) or plant density (Experiments 2 and 3) to create a wide range of N availability conditions. Table 5 provides a summary of the experimental conditions for each field trial, including planting dates, plant densities, N rates, N timing applications, and plot sizes. Out of the three experiments, only Experiment 1 also received 17 kg N ha^−1^ and 6 kg P ha^−1^ as band-applied starter fertilizer (19-17-0), and supplementary sprinkler irrigation (through a center pivot system) when needed. In all three experiments, plots were kept weed-free and pesticide management practices followed Purdue University recommendations.

### 4.2. Measurements and Calculations

Over the course of the growing season, phenological stages were recorded according to the scale by Ritchie and Hanway [62]. Stages were determined based on the day when 50% of 20 previously marked plants per plot reached that stage. At V4 stage, plant populations were determined by counting four sub-areas for each plot. Additionally, another 60 plants per plot (from the respective center pair of rows in each experiment) were tagged around V12 stage, and their respective silking dates were recorded individually. Phenology records were accompanied by weather data obtained for each growing season. Data from two nearby weather stations (“Wanatah 2 WNW”, La Porte, IN and “Knox WWTP”, Starke, IN; [63]) were averaged to account for Experiment 1. For Experiments 2 and 3, weather data were retrieved from the on-site station (“ACRE-West Lafayette”) [64]. Data obtained for each season included air temperature (maximum and minimum) and precipitation (Table 6). 

Above-ground biomass was sampled at silking (R1 stage), and at the onset (R3 stage) plus the end (R6 stage) of the effective grain-filling period. In each sampling, 10 plants were cut off at ground level from center rows in each plot. Plants were separated into their different components (leaf plus husks, stem plus tassels, ear, cob, grain), dried at 60 °C to constant weight, and weighed to determine component dry matters. After weighing, samples were ground (to pass a 1 mm sieve) and sent for N concentration analysis by combustion methods [65] to A&L Laboratories, IN (Exp. 1) and to Ward Laboratories, NE (Exp. 2 and 3). To ensure data consistency between labs, backup sub-samples of ground plant tissue from 2017 were submitted to Ward Laboratories and vice versa, backup ground material from 2018/2019 was submitted to A&L Laboratories. Reported concentrations from duplicate samples were similar. At each sampling stage, total-plant growth or total-plant N uptake was calculated by adding up DM or N content from all the respective separate components. Plant growth and plant N uptake during the whole grain-filling period were calculated by subtracting total-plant DM and N between R6 and R3 stages. Additionally, nitrogen nutrition index (NNI, [39]) was calculated on a whole-plant, per unit area, basis at both R1 and R3 stages by following Equation (1):

(1) $$\mathrm{NNI}\;=\frac{{\%\;N}_{a}}{{\%\;N}_{c}}$$

where %N_a_ is the whole-plant N concentration at either R1 or R3 stage and %N_c_ is the critical N concentration for that biomass. %N_c_ was obtained by applying the coefficients proposed for maize by Plénet and Lemaire [54] on each plot’s whole-plant biomass (W, Mg ha^−1^) for each stage as follows:

(2) $$\%\;N_{c}=3.4\;W^{-0.37}$$

To account for grain-filling dynamics, an intense ear sampling was performed. Beginning at R3 stage (12−17 DAS), four ears from previously tagged plants (i.e., of known silking date) were sampled from each plot on a weekly basis (i.e., 7−8 days apart). Overall, there were nine (Experiments 1 and 2) and ten (Experiment 3) sampling dates (i.e., weeks), thus making a total of 2796 ears processed: 972 in Experiment 1, 864 in Experiment 2, and 960 in Experiment 3. From the center section of each ear, 15 intact kernels were collected, weighed immediately after (i.e., fresh weight), and then weighed again after drying for 24 h at 100 °C (i.e., dry weight). An additional grain sub-sample was taken from the same ears once they were dried until constant weight at 60 °C. A composite sample was formed with the four ears of the same plot. From this composite sample, kernels were ground and analyzed for N concentration following the same procedure as that of the other plant tissue samples explained above. Therefore, for each plot at each sampling date, there were four DM values, and only one N concentration value. Kernel N content was calculated by multiplying the plot N concentration value over the four DM points, thus obtaining four N content values per plot per sampling date.

For each experiment, kernel DM and N accumulation data on a thermal time (TT, °C day) basis was fit using the linear-plateau model defined by Equations (3) and (4):

(3) KW or KNC=a+b TT,  when TT ≤ c

(4) KW or KNC=a+bc,                when TT>c

where *a* is the *y*-intercept (mg), *b* is the rate of the grain filling (EGFR, mg °C day^−1^) or the rate of N accumulation in grain (KNAR, mg N °C day^−1^), and *c* is the total duration of grain filling (GFD, °C day) or the total duration of N accumulation in grain (KNAD, °C day). Thermal time accumulation started at silking of each plant, using the average of maximum and minimum daily air temperature and a base temperature of 0 °C [66].

Grain yield (GY) was determined from different plants. For Exp. 1, GY was obtained by an eight row combine, harvesting the eight center rows of each plot. Yield monitor data were corrected by cropping 21 m of border at both ends of the original plot length in order to only consider the yield points recorded when the combine reached a stable harvesting flow. For Exp. 2 and 3, GY was hand-harvested from a 3 m^2^ plot area that was properly bordered. In all experiments, GY was expressed on a per area basis and adjusted to 15.5% moisture content. Kernel number (KN) and kernel weight (KW) were both estimated from the R6 biomass sample. KN resulted from counting all kernels in the 10-ear R6 sample, whereas five sub-samples of 200 kernels per plot were weighed to estimate KW.

### 4.3. Statistical Analysis

All analyses were conducted using R programming language [67]. Treatment effects on the parameters of the linear-plateau models fit to the KW and KNC data were tested using non-linear, mixed-effects models via the function nlme() within the package *nlme* [68]. Final models of both KW and KNC vs TT included only N rate fixed effects on the parameters *a*, *b* and *c*. The fixed effects of N timing (Exp. 1) and plant density (Exp. 2 and 3) were also considered in the initial models (in all possible parameter combinations), though these factors were ultimately excluded due to issues of lack of convergence and, less frequently, a null improvement (or worsening) of the goodness of fit due to overparameterization. Comparison among models was based on the Akaike Information Criterion (AIC), for which the best model is that with the smallest AIC value [69]. The random effects for each parameter were modeled with the diagonal variance-covariance matrix. Differences among the estimated parameters due to N rate fixed effects were tested through pairwise contrasts with the function emmeans() within the package *emmeans* [70], which uses the Tukey method for *p*-value adjustment of a family of estimates (multiple testing). The argument “weights = varPower” was included to account for the heteroscedasticity caused by the increase in variance over time, and the corrections were evidenced by the corresponding residual analyses. Normality assumptions were always satisfied.

N rate, N timing application, plant density, and interaction effects on individual variables were tested by ANOVA, following the respective split-plot structure of each experiment (i.e., whole plots nested within blocks, subplots nested within whole plots). Means separation was tested by least significant difference (LSD) at α = 0.05. Both ANOVA and LSD were conducted using the package *agricolae* [71]. Finally, relationships between parameters were studied via Pearson’s correlation analysis.

## 5. Conclusions

We were able to characterize N accumulation dynamics in maize kernels via the estimation of kernel N accumulation rate (KNAR) and duration (KNAD) on a thermal time basis for the first time. Alongside estimations of the more common kernel DM parameters (i.e., effective grain filling rate (EGFR) and grain filling duration (GFD)), we uncovered some intertwined interactions between DM and N dynamics during the grain fill period. Kernels actively accumulated N until late in the season, as shown by the similar GFD and KNAD values reached, as well as their high correlation. While EGFR was less impacted by N rate differences, KNAR was much responsive, showing a strong correlation with final KW. Increases in N supply, regardless of application timing or plant density, changed kernel DM accumulation by either increasing both EGFR and GFD, or by increasing GFD alone. In turn, both KW and kernel N accumulation at maturity increased consistently under higher N availability because of gains in both KNAR and KNAD. Whole-plant N status just before the linear period of grain fill (NNIR3) was important to subsequent whole-plant N uptake plus final grain yield and kernel N content, but the actual rates and duration of kernel DM filling were more strongly associated with KNAR than with prior NNI. Progressively higher NNI at R1 and R3 in response to N rates apparently improved KNAD and KNAR, but whole-plant NNI at these reproductive stages had less influence on kernel DM accumulation after R3 than the simultaneous accumulation of kernel N. Our results confirm a direct role of kernel N accumulation in limiting kernel DM allocation, and therefore final KW, during grain filling.

## Figures and Tables

**Table 1:** ANOVA for grain yield (GY, Mg ha^−1^, 15.5% moisture), kernel number per plant (KNP, grain plant^−1^), kernel weight (KW, mg grain^−1^), kernel N concentration (KNc, %), kernel N content (KNC, mg N grain^−1^), plant growth at R3 (PG_R3_, g plant^−1^), plant growth during the effective grain filling period (PG_R3_._R6_, g plant^−1^), plant N uptake at R3 (PNU_R3_, g N plant^−1^), plant N uptake during the effective grain filling (PNU_R3_._R6_, g N plant^−1^), NNI at silking (NNI_R1_, dimensionless), and NNI at R3 (NNI_R3_, dimensionless) in Experiment 1 (La Crosse, IN, 2017).

	GY	KNP	KW	KNc	KNC	PG_R3_	PG_R3_._R6_	PNU_R3_	PNU_R3_._R6_	NNI_R1_	NNI_R3_
**N Timing Application**											
Planting	10.5	483	261.1	1.05	2.82	170	61	1.58	0.47	0.94	0.69
Planting_V6	11.3	468	274.4	1.08	3.05	168	71	1.64	0.56	0.94	0.73
Planting_V12	11.2	492	263.4	1.08	2.92	166	65	1.60	0.50	0.99	0.72
**N Rate (kg N ha^−1^)**											
0 N	5.3 c	314 b	213.3 c	0.80 c	1.71 c	124 b	15 c	0.72 c	0.15 c	0.57 c	0.40 c
112 N	11.9 b	560 a	271.3 b	1.11 b	3.02 b	186 a	78 b	1.85 b	0.43 b	1.05 b	0.80 b
224 N	15.7 a	569 a	314.3 a	1.29 a	4.07 a	193 a	103 a	2.24 a	0.96 a	1.24 a	0.94 a
**F-test**											
N Timing (T)	ns	ns	ns	ns	ns	ns	ns	ns	ns	ns	ns
N Rate (N)	<0.001	<0.001	<0.001	<0.001	<0.001	<0.001	<0.001	<0.001	<0.001	<0.001	<0.001
T × N	ns	ns	ns	ns	ns	ns	ns	ns	ns	ns	ns

ns: not significant at α = 0.05, *p*-value for F-test is >0.05. Means separation determined by Fisher’s least significant difference (LSD) at α = 0.05. Same letter or absence of letter means no significant difference was found among levels. For all variables, three replicates were collected (*n* = 3).

**Table 2:** ANOVA for grain yield (GY, Mg ha^−1^, 15.5% moisture), kernel number per plant (KNP, grain plant^−1^), kernel weight (KW, mg grain^−1^), kernel N concentration (KNc, %), kernel N content (KNC, mg N grain^−1^), plant growth at R3 (PG_R3_, g plant^−1^), plant growth during the effective grain filling period (PG_R3_._R6_, g plant^−1^), plant N uptake at R3 (PNU_R3_, g N plant^−1^), plant N uptake during the effective grain filling (PNU_R3_._R6_, g N plant^−1^), NNI at silking (NNI_R1_, dimensionless), and NNI at R3 (NNI_R3_, dimensionless) in Experiment 2 (West Lafayette, IN, 2018).

	GY	KNP	KW	KNc	KNC	PG_R3_	PG_R3_._R6_	PNU_R3_	PNU_R3_._R6_	NNI_R1_	NNI_R3_
**N Rate (kg N ha^−1^)**											
0 N	7.4 d	255 c	267.7 b	0.86 c	2.29 c	119 c	29 b	0.77 c	0.13 b	0.43 c	0.44 c
84 N	11.4 c	356 b	283.9 b	0.95 b	2.70 b	143 b	54 b	1.20 b	0.20 b	0.70 b	0.63 b
168 N	15.3 b	475 a	309.6 a	1.13 a	3.50 a	164 ab	87 a	1.94 a	0.28 b	1.01 a	0.97 a
224 N	16.6 a	505 a	318.3 a	1.15 a	3.65 a	165 a	110 a	2.03 a	0.64 a	1.08 a	0.95 a
**Plant Density (plant m^−2^)**											
7.9 D	12.8	455 a	301.7 a	1.03	3.13	163 a	86 a	1.70 a	0.37	0.82	0.77
10.4 D	12.5	340 b	288.0 b	1.01	2.95	131 b	54 b	1.26 b	0.25	0.79	0.73
**F-test**											
N Rate (N)	<0.001	<0.001	0.002	<0.001	<0.001	0.005	0.004	<0.001	0.026	<0.001	<0.001
Density (D)	ns	<0.001	0.010	ns	ns	<0.001	0.001	0.002	ns	ns	ns
N × D	ns	ns	ns	ns	ns	ns	ns	ns	ns	ns	ns

ns: not significant at α = 0.05, *p*-value for F-test is >0.05. Means separation determined by Fisher’s least significant difference (LSD) at α = 0.05. Same letter or absence of letter means no significant difference was found among levels. For all variables, three replicates were collected (*n* = 3).

**Table 3:** ANOVA for grain yield (GY, Mg ha^−1^, 15.5% moisture), kernel number per plant (KNP, grain plant^−1^), kernel weight (KW, mg grain^−1^), kernel N concentration (KNc, %), kernel N content (KNC, mg N grain^−1^), plant growth at R3 (PG_R3_, g plant^−1^), plant growth during the effective grain filling period (PG_R3_._R6_, g plant^−1^), plant N uptake at R3 (PNU_R3_, g N plant^−1^), plant N uptake during the effective grain filling (PNU_R3_._R6_, g N plant^−1^), NNI at silking (NNI_R1_, dimensionless), and NNI at R3 (NNI_R3_, dimensionless) in Experiment 3 (West Lafayette, IN, 2019).

	GY	KNP	KW	KNc	KNC	PG_R3_	PG_R3_._R6_	PNU_R3_	PNU_R3_._R6_	NNI_R1_	NNI_R3_
**N Rate (kg N ha^−1^)**											
0 N	7.5 c	288 c	264.9 c	0.88 c	2.34 c	127 b	26 b	0.88 b	0.10	0.57 c	0.50 b
84 N	9.5 b	342 b	282.3 b	0.95 bc	2.68 bc	131 b	47 a	1.06 b	0.20	0.74 b	0.60 b
168 N	11.0 ab	406 a	295.3 ab	1.06 ab	3.12 ab	169 a	47 a	1.66 a	0.19	0.94 a	0.77 a
224 N	12.2 a	424 a	299.2 a	1.16 a	3.48 a	167 a	60 a	1.84 a	0.20	0.98 a	0.87 a
**Plant Density (plant m^−2^)**											
7.9 D	10.0	405 a	287.6	1.03	2.97 a	162 a	50	1.51 a	0.20	0.82	0.69
10.4 D	10.1	325 b	283.2	1.00	2.84 b	135 b	39	1.21 b	0.14	0.80	0.68
**F-test**											
N Rate (N)	0.004	<0.001	0.008	0.008	0.004	<0.001	0.011	0.002	ns	0.002	0.006
Density (D)	ns	<0.001	ns	ns	0.012	<0.001	ns	0.012	ns	ns	ns
N × D	0.024	ns	ns	ns	ns	ns	ns	ns	ns	ns	ns

ns: not significant at α = 0.05, *p*-value for F-test is >0.05. Means separation determined by Fisher’s least significant difference (LSD) at α = 0.05. Same letter or absence of letter means no significant difference was found among levels. For all variables, three replicates were collected (*n* = 3).

**Table 4:** Correlation analysis between physiological parameters obtained in Exp. 1, 2 and 3 (*n* = 11). Data included: a) main N rate means per year of grain yield (GY, Mg ha^−1^, 15.5% moisture), kernel number per plant (KNP, grain plant^−1^), final kernel weight (KW, mg grain^−1^), final kernel N content (KNC, mg N grain^−1^), plant growth at R3 (PG_R3_, g plant^−1^), plant growth during the effective grain filling period (PG_R3_._R6_, g plant^−1^), plant N uptake at R3 (PNU_R3_, g N plant^−1^), plant N uptake during the effective grain filling (PNU_R3_._R6_, g N plant^−1^), NNI at silking (NNI_R1_, dimensionless), and NNI at R3 (NNI_R3_, dimensionless); (b) coefficients of effective grain-filling rate (EGFR, mg °C day^−1^), grain-filling duration (GFD, °C day), kernel N accumulation rate (KNAR, mg N °C day^−1^), and kernel N accumulation duration (KNAD, °C day) estimated for each N rate treatment per year. Pairwise Pearson’s coefficients (r) are located above the diagonal. Significance results are located below the diagonal.

	GY	KNP	KW	KNC	EGFR	GFD	KNAD	KNAR	PG_R3_	PG_R3_._R6_	PNU_R3_	PNU_R3_._R6_	NNI_R1_	NNI_R3_
GY		0.84	0.90	0.95	0.74	0.69	0.86	0.83	0.80	0.97	0.93	0.74	0.89	0.96
KNP	**		0.61	0.83	0.42	0.36	0.77	0.60	0.95	0.90	0.94	0.81	0.96	0.88
KW	***	*		0.92	0.93	0.89	0.87	0.95	0.65	0.82	0.81	0.56	0.73	0.86
KNC	***	**	***		0.80	0.73	0.92	0.92	0.86	0.91	0.96	0.75	0.92	0.96
EGFR	**	ns	***	**		0.84	0.71	0.92	0.52	0.61	0.64	0.38	0.57	0.71
GFD	*	ns	***	*	**		0.83	0.88	0.42	0.59	0.60	0.25	0.51	0.69
KNAD	***	**	***	***	*	**		0.88	0.79	0.83	0.89	0.60	0.86	0.90
KNAR	**	*	***	***	***	***	***		0.69	0.72	0.82	0.48	0.77	0.87
PG_R3_	**	***	*	***	ns	ns	**	*		0.81	0.95	0.73	0.96	0.88
PG_R3_._R6_	***	***	**	***	*	.	**	*	**		0.92	0.83	0.89	0.92
PNU_R3_	***	***	**	***	*	.	***	**	***	***		0.75	0.98	0.98
PNU_R3_._R6_	**	**	.	**	ns	ns	*	ns	*	**	**		0.78	0.66
NNI_R1_	***	***	*	***	.	ns	***	**	***	***	***	**		0.94
NNI_R3_	***	***	***	***	*	*	***	***	***	***	***	*	***	

(ns): *p* > 0.1; (.): *p* < 0.1; (*): *p* < 0.05; (**): *p* < 0.01; (***): *p* < 0.001.

**Table 5:** Characteristics of field experiments. Each experiment was conducted under a split-plot arrangement of treatments. Respective whole plot and sub-plot factors are detailed for each experiment.

Study	Location	Planting Date	Plant Density	N Rate	N Timing Application	Plot Size	Reps
Exp. 1	La Crosse, IN	16 May 2017	8.3 plants m^−2^	*Sub-plot* : 0 112 kg N ha^−1^224 kg N ha^−1^	*Whole plot:* At planting Split between planting and V6 Last 56 kg N ha^−1^ at V12	12 rows 0.76 m row width228 m row length	3
Exp. 2	West Lafayette, IN	8 May 2018	*Sub-plot:* 7.9 plants m^−2^ 10.4 plants m^−2^	*Whole plot:* 0 84 kg N ha^−1^ 168 kg N ha^−1^ 224 kg N ha^−1^	At planting	4 rows 0.76 m row width 14.5 m row length	3
Exp. 3	West Lafayette, IN	3 June 2019	*Sub-plot:* 7.9 plants m^−2^ 10.4 plants m^−2^	*Whole plot:* 0 84 kg N ha^−1^ 168 kg N ha^−1^ 224 kg N ha^−1^	At planting	4 rows 0.76 m row width 14.5 m row length	3

**Table 6:** Weather conditions between key growth stages for each field experiment.

Climate Parameter	Growth Stage Interval	Experiment 1	Experiment 2	Experiment 3
Mean Minimum Temperature (°C)	P-V12	13.3	16.2	17.5
	V12-R1	16.1	18.8	15.0
	R1-R3	15.5	16.9	16.4
	R3-R6	11.7	17.0	13.8
Mean Maximum Temperature (°C)	P-V12	26.6	27.6	28.3
	V12-R1	26.8	29.2	27.6
	R1-R3	27.2	28.2	28.8
	R3-R6	25.1	27.3	26.1
Cumulative Rainfall (mm)	P-V12	201	201	127
	V12-R1	79	48	4
	R1-R3	96	14	29
	R3-R6	84 *	164	105

*: 59 mm of rainfall plus 25 mm of irrigation.

## Data Availability

Data will be made available by the authors upon written request. The Ph.D. thesis data summarized here are presently being utilized as background support for conclusions (being written in another manuscript) from other data parameters that were simultaneously collected from these field studies.

## Supplementary Materials

The following are available online at https://www.mdpi.com/article/10.3390/plants10061222/s1. **Table S1**: N rate treatment comparison for parameters *a*, *b* (EGFR) and *c* (GFD) in Experiment 1 (La Crosse, IN, 2017). For each parameter, contrasts’ *p*-values were adjusted by Tukey method for comparing a family of 3 estimates. **Table S2**: N rate treatment comparison for parameters *a*, *b* (EGFR) and *c* (GFD) in Experiment 2 (West Lafayette, IN, 2018). For each parameter, contrasts’ *p*-values are adjusted by Tukey method for comparing a family of 4 estimates. **Table S3**. N rate treatment comparison for parameters *a*, *b* (EGFR) and *c* (GFD) in Experiment 3 (West Lafayette, IN, 2019). For each parameter, contrasts’ *p*-values are adjusted by Tukey method for comparing a family of 4 estimates. **Table S4**. N rate treatment comparison for parameters *a*, *b* (KNAR) and *c* (KNAD) in Experiment 1 (La Crosse, IN, 2017). For each parameter, contrasts’ *p*-values were adjusted by Tukey method for comparing a family of 3 estimates. **Table S5**. N rate treatment comparison for parameters *a*, *b* (KNAR) and *c* (KNAD) in Experiment 2 (West Lafayette, IN, 2018). For each parameter, contrasts’ *p*-values are adjusted by Tukey method for comparing a family of 4 estimates. **Table S6**. N rate treatment comparison for parameters *a*, *b* (KNAR) and *c* (KNAD) in Experiment 3 (West Lafayette, IN, 2019). For each parameter, contrasts’ *p*-values are adjusted by Tukey method for comparing a family of 4 estimates. **Figure S1**. Relationship between NNI estimated at R1 (light symbols) and at R3 (dark symbols) and kernel number. Each symbol represents data on a per plot basis from three different field experiments testing N timing application treatments (Exp. 1) and plant densities (Exp. 2 and 3) in combination with N rate treatments.
