# Supplementary material for: Dry Matter Gains in Maize Kernels Are Dependent on Their Nitrogen Accumulation Rates and Duration during Grain Filling
Source: Plants (Basel). 2021 Jun 15;10(6):1222. doi: 10.3390/plants10061222 (PMC8232743; doi:10.3390/plants10061222)
Supplement: Supplementary file 1 [file plants-10-01222-s001.zip › plants-1205074-supplementary.pdf]

Supplementary Materials

For Tables S1, S2 and S3:
Linear-plateau model for maize kernel weight (KW, mg grain^{-1}) versus thermal time (TT, ºC day) after silking:

$$KW = a + b\,TT, \qquad when\ TT \leq c$$
$$KW = a + bc, \qquad when\ TT > c$$

where a is the y-intercept (mg), b is the effective grain-filling rate (EGFR, mg ºC day^{-1}) and c is the grain-filling duration (GFD, ºC days).

Table S1. N rate treatment comparison for parameters a, b (EGFR) and c (GFD) in Experiment 1 (La Crosse, IN, 2017). For each parameter, contrasts' p-values were adjusted by Tukey method for comparing a family of 3 estimates.

**Parameter**	**Contrast**	**Estimate**	**Standard Error**	**Degrees of Freedom**	**t ratio**	**p-value**
a	0N - 112N	17.9	5.27	24	3.393	0.0065
	0N - 224N	33.4	5.22	24	6.392	< 0.0001
	112N - 224N	15.5	5.78	24	2.68	0.0338
**EGFR (b)**	0N - 112N	-0.0362	0.0064	906	-5.663	< 0.0001
	0N - 224N	-0.0697	0.00633	906	-11.016	< 0.0001
	112N - 224N	-0.0335	0.00684	906	-4.892	< 0.0001
**GFD (c)**	0N - 112N	-116.3	24.1	906	-4.835	< 0.0001
	0N - 224N	-143.5	24	906	-5.967	< 0.0001
	112N - 224N	-27.2	24	906	-1.132	0.4943

Table S2. N rate treatment comparison for parameters *a*, *b* (EGFR) and *c* (GFD) in Experiment 2 (West Lafayette, IN, 2018). For each parameter, contrasts' p-values are adjusted by Tukey method for comparing a family of 4 estimates.

Parameter	Contrast	Estimate	Standard Error	Degrees of Freedom	t ratio	p-value
a	0N - 84N	10.37	3.63	20	2.856	0.0445
	0N - 168N	12.8	3.7	20	3.458	0.0122
	0N - 224N	8.06	3.6	20	2.242	0.146
	84N - 168N	2.44	4	20	0.609	0.928
	84N - 224N	-2.31	3.9	20	-0.591	0.9337
	168N - 224N	-4.74	3.97	20	-1.195	0.6371
**EGFR (*b*)**	0N - 84N	-0.0203	0.00564	822	-3.596	0.002
	0N - 168N	-0.02832	0.0055	822	-5.153	< 0.0001
	0N - 224N	-0.01901	0.00526	822	-3.614	0.002
	84N - 168N	-0.00802	0.00599	822	-1.339	0.538
	84N - 224N	0.00129	0.00577	822	0.224	0.996
	168N - 224N	0.00932	0.00563	822	1.655	0.3485
**GFD (*c*)**	0N - 84N	9.55	17.7	822	0.538	0.9496
	0N - 168N	-44.24	17.7	822	-2.5	0.0606
	0N - 224N	-91.36	17.9	822	-5.092	< 0.0001
	84N - 168N	-53.8	17.6	822	-3.065	0.012
	84N - 224N	-100.91	17.8	822	-5.669	< 0.0001
	168N - 224N	-47.11	17.8	822	-2.654	0.0404

Table S3. N rate treatment comparison for parameters *a*, *b* (EGFR) and *c* (GFD) in Experiment 3 (West Lafayette, IN, 2019). For each parameter, contrasts' p-values are adjusted by Tukey method for comparing a family of 4 estimates.

Parameter	Contrast	Estimate	Standard Error	Degrees of Freedom	t ratio	p-value
a	0N - 84N	-2.369	3.36	20	-0.705	0.8939
	0N - 168N	1.662	3.41	20	0.487	0.961
	0N - 224N	0.933	3.52	20	0.265	0.9933
	84N - 168N	4.031	3.34	20	1.208	0.6293
	84N - 224N	3.302	3.45	20	0.957	0.7748
	168N - 224N	-0.729	3.5	20	-0.208	0.9967
**EGFR (*b*)**	0N - 84N	0.00229	0.00611	914	0.374	0.9821
	0N - 168N	-0.01024	0.00611	914	-1.674	0.338
	0N - 224N	-0.00757	0.00626	914	-1.21	0.6208
	84N - 168N	-0.01252	0.00597	914	-2.097	0.155
	84N - 224N	-0.00986	0.00612	914	-1.611	0.373
	168N - 224N	0.00267	0.00613	914	0.435	0.9724
**GFD (*c*)**	0N - 84N	-19.1	22	914	-0.869	0.8206
	0N - 168N	-37.3	21.9	914	-1.703	0.3227
	0N - 224N	-56.5	22	914	-2.564	0.0513
	84N - 168N	-18.2	21.9	914	-0.831	0.8396
	84N - 224N	-37.3	22	914	-1.698	0.3254
	168N - 224N	-19.1	21.9	914	-0.874	0.8185

For Tables S4, S5 and S6:
Linear-plateau model for maize kernel N content (KNC, mg N grain^{-1}) versus thermal time (TT, ºC day) after silking:

$$KNC = a + b\,TT, \qquad when\ TT \leq c$$
$$KNC = a + bc, \qquad when\ TT > c$$

where *a* is the *y*-intercept (mg), *b* is the kernel N accumulation rate (KNAR, mg N ºC day^{-1}) and *c* is the kernel N accumulation duration (KNAD, ºC days).

Table S4. N rate treatment comparison for parameters *a*, *b* (KNAR) and *c* (KNAD) in Experiment 1 (La Crosse, IN, 2017). For each parameter, contrasts' p-values were adjusted by Tukey method for comparing a family of 3 estimates.

Parameter	Contrast	Estimate	Standard Error	Degrees of Freedom	t ratio	p-value
a	0N - 112N	0.193	0.0604	24	3.195	0.0105
	0N - 224N	0.608	0.0643	24	9.459	< 0.0001
	112N - 224N	0.415	0.0765	24	5.43	< 0.0001
KNAR (*b*)	0N - 112N	-0.00112	9.14E-05	906	-12.246	< 0.0001
	0N - 224N	-0.00211	0.000101	906	-20.917	< 0.0001
	112N - 224N	-0.00099	0.000115	906	-8.543	< 0.0001
KNAD (*c*)	0N - 112N	-122	24.1	906	-5.061	< 0.0001
	0N - 224N	-142.6	23.7	906	-6.003	< 0.0001
	112N - 224N	-20.6	24.7	906	-0.836	0.6808

Table S5. N rate treatment comparison for parameters *a*, *b* (KNAR) and *c* (KNAD) in Experiment 2 (West Lafayette, IN, 2018). For each parameter, contrasts' p-values are adjusted by Tukey method for comparing a family of 4 estimates.

Parameter	Contrast	Estimate	Standard Error	Degrees of Freedom	t ratio	p-value
a	0N - 84N	0.0748	0.0422	20	1.775	0.314
	0N - 168N	0.2879	0.0474	20	6.074	< 0.0001
	0N - 224N	0.2431	0.045	20	5.396	0.0002
	84N - 168N	0.2131	0.0521	20	4.087	0.003
	84N - 224N	0.1683	0.05	20	3.365	0.015
	168N - 224N	-0.0448	0.0545	20	-0.822	0.8434
KNAR (*b*)	0N - 84N	-0.00032	7.46E-05	822	-4.329	0.0001
	0N - 168N	-0.00117	8.03E-05	822	-14.566	< 0.0001
	0N - 224N	-0.00107	7.47E-05	822	-14.269	< 0.0001
	84N - 168N	-0.00085	8.66E-05	822	-9.771	< 0.0001
	84N - 224N	-0.00074	8.15E-05	822	-9.118	< 0.0001
	168N - 224N	0.000103	8.67E-05	822	1.193	0.6315
KNAD (*c*)	0N - 84N	-21.9	33.6	822	-0.653	0.9147
	0N - 168N	-66	33.2	822	-1.989	0.1929
	0N - 224N	-124.7	33.4	822	-3.733	0.0012
	84N - 168N	-44.1	33.1	822	-1.33	0.5438
	84N - 224N	-102.8	33.4	822	-3.081	0.0114
	168N - 224N	-58.7	32.9	822	-1.783	0.2824

Table S6. N rate treatment comparison for parameters *a*, *b* (KNAR) and *c* (KNAD) in Experiment 3 (West Lafayette, IN, 2019). For each parameter, contrasts' p-values are adjusted by Tukey method for comparing a family of 4 estimates.

Parameter	Contrast	Estimate	Standard Error	Degrees of Freedom	t ratio	p-value
a	0N - 84N	0.0425	0.029	20	1.467	0.4747
	0N - 168N	0.2003	0.0302	20	6.64	< 0.0001
	0N - 224N	0.1813	0.032	20	5.658	0.0001
	84N - 168N	0.1577	0.0295	20	5.345	0.0002
	84N - 224N	0.1388	0.0314	20	4.416	0.0014
	168N - 224N	-0.0189	0.0325	20	-0.583	0.9361
**KNAR (*b*)**	0N - 84N	-0.00024	8.37E-05	914	-2.808	0.0261
	0N - 168N	-0.00079	8.49E-05	914	-9.255	< 0.0001
	0N - 224N	-0.00082	8.65E-05	914	-9.503	< 0.0001
	84N - 168N	-0.00055	8.45E-05	914	-6.522	< 0.0001
	84N - 224N	-0.00059	0.000086	914	-6.82	< 0.0001
	168N - 224N	-3.6E-05	8.73E-05	914	-0.409	0.9768
**KNAD (*c*)**	0N - 84N	-39.4	28.3	914	-1.389	0.5065
	0N - 168N	-49.5	28.1	914	-1.761	0.2928
	0N - 224N	-98.5	28.3	914	-3.477	0.003
	84N - 168N	-10.1	28	914	-0.36	0.9841
	84N - 224N	-59.1	28.3	914	-2.091	0.1566
	168N - 224N	-49	28	914	-1.751	0.2978

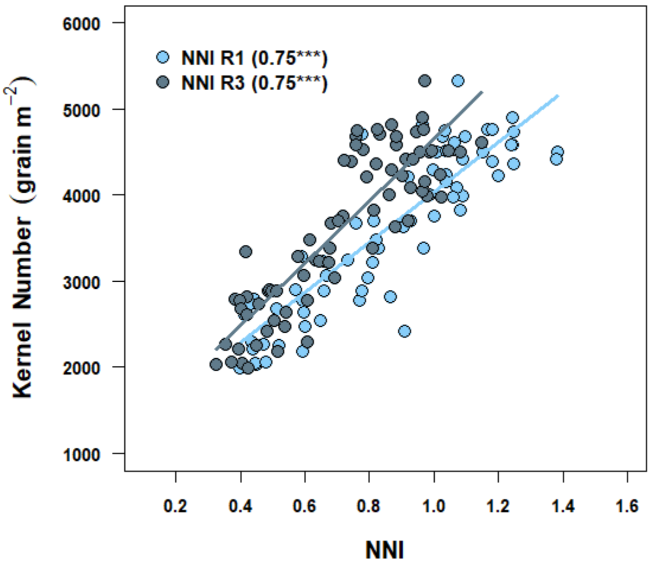

Figure S1. Relationship between NNI estimated at R1 (light symbols) and at R3 (dark symbols) and kernel number. Each symbol represents data on a per plot basis from three different field experiments testing N timing application treatments (Exp. 1) and plant densities (Exp. 2 and 3) in combination with N rate treatments.
